# Supplementary material for: An unconventional gatekeeper mutation sensitizes inositol hexakisphosphate kinases to an allosteric inhibitor
Source: eLife. 2023 Oct 16;12:RP88982. doi: 10.7554/eLife.88982 (PMC10578927; doi:10.7554/eLife.88982)
Supplement: Figure 4—figure supplement 1—source data 1. [file elife-88982-fig4-figsupp1-data1.docx]

**Supporting Information Table. Data collection and structure refinement statistics**

|  | *Entamoeba histolytica* IP6KA M85V variant |
| --- | --- |
| PDB ID code  **Data collection** | 8OMI |
| Space group | I4_1_22 |
| Cell dimensions |  |
| *a*, *b*, *c* (Å) | 102.82, 102.82, 111.61 |
| *α*, *β*, *γ* (°) | 90, 90, 90 |
| Resolution (Å) | 37.81-1.77 (1.84-1.77)* |
| *R*_merge_ (%) | 12.51 (251.1) |
| < *I* / σ(*I*) > | 8.61 (1.11) |
| Completeness (%) | 99.15 (98.25) |
| Redundancy | 7.5 (7.6) |
|  |  |
| **Refinement** |  |
| Resolution (Å) | 1.77 |
| No. unique reflections | 29205 |
| *R*_work_ / *R*_free_ (%) | 23.08 / 25.36 |
| No. non-hydrogen atoms | 2233 |
| Protein  Ligands | 1999  45 |
| Water | 189 |
| Average B-factor (Å²) |  |
| Overall | 39.2 |
| Protein  Ligands | 38.6  49.7 |
| Water | 42.5 |
| R.m.s deviations |  |
| Bond lengths (Å) | 0.002 |
| Bond angles (º) | 0.541 |

*Values in parenthesis are for highest resolution shell.

One single crystal was used to collect a complete dataset for structure determination.
